# #4Corners4Health Social Media Cancer Prevention Campaign for Emerging Adults: Protocol for a Randomized Stepped-Wedge Trial

**DOI:** 10.2196/50392

**Published:** 2024-02-22

**Authors:** David B Buller, Andrew L Sussman, Cynthia A Thomson, Deanna Kepka, Douglas Taren, Kimberly L Henry, Echo L Warner, Barbara J Walkosz, W Gill Woodall, Kayla Nuss, Cindy K Blair, Dolores D Guest, Evelinn A Borrayo, Judith S Gordon, Jennifer Hatcher, David W Wetter, Alishia Kinsey, Christopher F Jones, Angela K Yung, Kaila Christini, Julia Berteletti, John A Torres, Emilia Yessenya Barraza Perez, Annelise Small

**Affiliations:** 1 Klein Buendel Golden, CO United States; 2 University of New Mexico Comprehensive Cancer Care Center Albuquerque, NM United States; 3 Department of Health Promotion Sciences Mel & Enid Zuckerman College of Public Health University of Arizona Tucson, AZ United States; 4 College of Nursing and Huntsman Cancer Institute University of Utah Salt Lake City, UT United States; 5 Section of Nutrition University of Colorado Denver Aurora, CO United States; 6 Department of Psychology College of Natural Sciences Colorado State University Fort Collins, CO United States; 7 University of Colorado Cancer Center University of Colorado Denver Aurora, CO United States; 8 College of Nursing University of Arizona Tucson, AZ United States; 9 Cancer Center University of Arizona Tucson, AZ United States; 10 Department of Population Health Sciences University of Utah Salt Lake City, UT United States; 11 Huntsman Cancer Institute University of Utah Salt Lake City, UT United States; 12 College of Medicine University of Arizona Tucson, AZ United States; 13 Department of Medicine University of Arizona Cancer Center Tucson, AZ United States

**Keywords:** cancer prevention, young adults, rural, social media, physical activity, diet, alcohol, tobacco control, sunburn, human papillomavirus, HPV vaccination

## Abstract

**Background:**

Many emerging adults (EAs) are prone to making unhealthy choices, which increase their risk of premature cancer morbidity and mortality. In the era of social media, rigorous research on interventions to promote health behaviors for cancer risk reduction among EAs delivered over social media is limited. Cancer prevention information and recommendations may reach EAs more effectively over social media than in settings such as health care, schools, and workplaces, particularly for EAs residing in rural areas.

**Objective:**

This pragmatic randomized trial aims to evaluate a multirisk factor intervention using a social media campaign designed with community advisers aimed at decreasing cancer risk factors among EAs. The trial will target EAs from diverse backgrounds living in rural counties in the *Four Corners* states of Arizona, Colorado, New Mexico, and Utah.

**Methods:**

We will recruit a sample of EAs (n=1000) aged 18 to 26 years residing in rural counties (Rural-Urban Continuum Codes 4 to 9) in the Four Corners states from the Qualtrics’ research panel and enroll them in a randomized stepped-wedge, quasi-experimental design. The inclusion criteria include English proficiency and regular social media engagement. A social media intervention will promote guideline-related goals for increased physical activity, healthy eating, and human papillomavirus vaccination and reduced nicotine product use, alcohol intake, and solar UV radiation exposure. Campaign posts will cover digital and media literacy skills, responses to misinformation, communication with family and friends, and referral to community resources. The intervention will be delivered over 12 months in Facebook private groups and will be guided by advisory groups of community stakeholders and EAs and focus groups with EAs. The EAs will complete assessments at baseline and at 12, 26, 39, 52, and 104 weeks after randomization. Assessments will measure 6 cancer risk behaviors, theoretical mediators, and participants’ engagement with the social media campaign.

**Results:**

The trial is in its start-up phase. It is being led by a steering committee. Team members are working in 3 subcommittees to optimize community engagement, the social media intervention, and the measures to be used. The Stakeholder Organization Advisory Board and Emerging Adult Advisory Board were formed and provided initial input on the priority of cancer risk factors to target, social media use by EAs, and community resources available. A framework for the social media campaign with topics, format, and theoretical mediators has been created, along with protocols for campaign management.

**Conclusions:**

Social media can be used as a platform to counter misinformation and improve reliable health information to promote health behaviors that reduce cancer risks among EAs. Because of the popularity of web-based information sources among EAs, an innovative, multirisk factor intervention using a social media campaign has the potential to reduce their cancer risk behaviors.

**Trial Registration:**

ClinicalTrials.gov NCT05618158; https://classic.clinicaltrials.gov/ct2/show/NCT05618158

**International Registered Report Identifier (IRRID):**

PRR1-10.2196/50392

## Introduction

### Background

Emerging adulthood is an important habit-forming period of life. The lives of emerging adults aged 18 to 26 years are in flux [1], as they experience lifestyle transitions and increased autonomy while taking on adult-related responsibilities (eg, financial, residential, and employment). It is an important time for health behaviors because with increased autonomy, numerous emerging adults are at risk of making unhealthy choices [2,3].

Health-compromising behaviors that increase cancer risk later in life are especially prevalent among emerging adults [4] and are linked to future cancer morbidity and mortality [5]. Many emerging adults have reduced physical activity and unhealthy eating patterns that do not meet the 2020 American Cancer Society guidelines [6] (are overweight, eat fast food [7,8], and have low self-efficacy for making healthy food choices) [9-13]. Emerging adults’ use of nicotine products [9-12,14] and multiple combustible and noncombustible products [15,16] and their alcohol use and heavy drinking (bingeing) are high [17,18]. Emerging adults are also more prone to intentional UV exposure (solar and artificial tanning) [19]; sporadic sun safety practices [20-23]; and a lack of human papillomavirus (HPV) knowledge [24,25], resulting in incomplete vaccination rates [26,27]. These modifiable cancer risk behaviors are important targets of primary prevention for emerging adults by promoting moderate to vigorous physical activity (MVPA) and dietary and sun safety skills, supporting the use of brief interventions for tobacco or alcohol use, and encouraging emerging adults to make their own health care decisions such as HPV vaccination. However, there is a lack of rigorous research on interventions for emerging adults, and information on their cancer risk behaviors is limited [28].

In the United States, rural populations have substantially higher rates of cancer [29] related to unhealthy eating [30,31], high rates of smoking and alcohol use, exposure to UV radiation and radon (riskier for smokers) [32,33], and persistent HPV infection compared with urban populations. Rural cancer disparities are exacerbated by a lack of health insurance and preventive care [34,35], low socioeconomic status [36,37], poor health literacy [36,38], fatalistic beliefs and ambiguous health information [25], and pervasive barriers to preventive health care [37,39,40].

Social media may offer a superior intervention channel for reaching and influencing emerging adults compared with health care, schools, and workplaces, including in rural areas. Emerging adults (90%) are the most engaged age group on the internet [41], and the internet is a preferred channel for health information among rural and urban emerging adults [40,42]. Rural adults use the internet (85%), as do most Hispanic (86%) and African American (85%) individuals [42]. Social media platforms are very popular with emerging adults [41,43]. Social media provide flexible, responsive, accessible, and low-cost platforms for distributing cancer information to the public from trusted voices [44]. Social media can improve information dissemination, credibility, and relevance and are often used to detect and respond to emerging issues [45] and promote engagement with personalized and impactful user-generated content [46]. Although use varies across platforms, there is growing evidence that interventions delivered using social media can improve physical activity, diet, nicotine product use, skin cancer prevention, and HPV vaccination outcomes among young adults [47-57]. By contrast, it can be challenging to implement a cancer prevention intervention in health care organizations, schools, and workplaces in low-resourced rural communities. Interventions in these channels also may not reach many emerging adults who have low preventive health care use, school enrollment, or employment. However, social media platforms also circulate inaccurate, misleading, and harmful information [58,59]. For instance, social media has spread misinformation on tobacco products [60], breast cancer prevention [61], the efficacy of cannabidiol for cancer care [62], cancer-related nutrition [63], and the mistrust of HPV vaccines [64,65], which can undermine cancer prevention efforts. Thus, interventions also need strategies to correct misinformation and provide digital and media literacy skills [66-70].

In summary, emerging adulthood is an important period for establishing cancer prevention. Many rural emerging adults experience several cancer risk factors, but successful interventions for them are lacking. With the popularity of web-based resources for emerging adults, an innovative multirisk factor intervention over social media has the potential to reach this underserved population and reduce emerging adults’ cancer risks. The goal of this trial is to modify cancer risk factors among emerging adults aged 18 to 26 years living in rural counties in the Four Corners states of Arizona, Colorado, New Mexico, and Utah using a unique, theory-based social media campaign designed with community advisers that delivers relevant, credible, and timely content on reducing multiple cancer risks to emerging adults. A multirisk factor approach [71,72] is adopted because (1) emerging adults vary in cancer risk profiles, (2) several risks cooccur and are affected by similar mechanisms [73-76], and (3) coverage of a variety of topics in the campaign will be engaging. Furthermore, the Four Corners region has a relatively high burden of poverty [77,78], significant population diversity with large Hispanic and American Indian populations [79], and low population density where distance and transportation are health care access barriers [37,80], providing a rich environment to test the efficacy of a multibehavior health promotion intervention.

### Objectives

Our proposed intervention will aim to aid rural emerging adults in making informed decisions to reduce cancer risks related to infrequent physical activity, unhealthy diet, alcohol intake (per 2020 American Cancer Society guidelines [6]), nicotine product use, UV exposure, and lack of HPV vaccination uptake. In addition, it will help them critically evaluate and resist misinformation and marketing that promote cancer and other health risk behaviors and support emerging adults to be media literate when using digital media. The trial will test the following hypotheses:

- Hypothesis 1: emerging adults will increase MVPA and healthy eating patterns, reduce nicotine product and alcohol use and sunburns, and increase HPV vaccine uptake from baseline to final assessment when receiving the social media intervention.
- Hypothesis 2: the positive impact of the social media campaign on cancer risk factors among emerging adults will be mediated by improved cancer risk knowledge and beliefs (ie, self-efficacy and response efficacy, norms, social support, and vaccine antecedents), digital and media literacy skills, misinformation identification, and family communication.

Analyses will also explore whether the impact of the campaign differs according to (1) the level of emerging adults’ engagement with it, (2) cancer risk factors, and (3) the biological sex of the participants. The prospective randomized quasi-experimental design and its large sample will provide a rigorous evaluation of the social media campaign compared with many previous studies on social media that have used less rigorous nonrandomized controlled trial designs and small samples [81-86].

## Methods

We will test a social media campaign to reduce cancer risk factors among emerging adults in rural counties in the Four Corners states using a randomized stepped-wedge trial design.

### Target Population and Recruitment Procedures

Emerging adults (N=1000) aged 18 to 26 years residing in rural counties in the states of Arizona, Colorado, New Mexico, and Utah will be enrolled in the study (refer to Textbox 1 for the inclusion and exclusion criteria). Many emerging adults will report ≥1 cancer risk behavior and will be residing in a variety of living arrangements, from multigenerational families to roommates to spouses or partners to alone. In the Four Corners states, 99 counties are rural (ie, Rural-Urban Continuum Codes [RUCC] 4 to 9 [87]), with >2 million residents (176,737 residents aged 19 to 25 years; annual income) [88]. Pregnant individuals will be excluded because the intervention will not provide individualized counseling and could lead to behavioral changes in diet or exercise that might be contraindicated during pregnancy.

**Inclusion and exclusion criteria for the sample of emerging adults.**

**Inclusion criteria**

- Member of the Qualtrics survey panel in year 2
- Aged 18 to 26 years
- Resides in a county coded as Rural-Urban Continuum Codes 4 to 9 in Arizona, Colorado, New Mexico, or Utah
- Able to speak and read English
- Has regular social media engagement
- Accepts screening call from the study staff
- Provides consent to participate

**Exclusion criteria**

- Participated in community engagement activities
- Cannot speak and read English
- Has low or no social media engagement
- Does not accept a screening call from the study staff
- Does not provide consent to participate
- Does not give permission for engagement data to be extracted from the Facebook private groups
- If biologically female, currently pregnant

Emerging adults will be recruited from Qualtrics’ research panel, built from multiple providers that use by-invitation or double opt-in methods. Qualtrics will select adults aged 18 to 26 years residing in counties designated as RUCC 4 to 9 in the 4 selected states [87], balanced on gender, and refer emerging adults to the project’s registration website to complete a consent form. Fake, duplicate, and unqualified respondents will be screened out, and steps will be taken to ensure that they participate only once. The project staff will contact the consented emerging adults by telephone and confirm their eligibility. To avoid clustering, only 1 emerging adult per household will be enrolled. If recruitment lags, we will add Mountain West states with similar populations (ie, Idaho, Montana, Nevada, and Wyoming). We will use quotas so that the sample matches the education and the race and ethnicity of the counties. Although research panel members are required to have internet access, selection bias will be reduced as most US adults (90%), including emerging adults (100%) and rural adults (85%), have access to the internet [42]. We will confirm that the emerging adults have regular social media use (post ≥1 time per week) to allow them to engage with our campaign. We acknowledge that this criterion may impact generalizability, but social media influence those who view them regularly [89,90]. The escalating compensation schedule is designed to achieve retention for postintervention assessments.

### Randomized Stepped-Wedge Trial Design

**Figure 1:**
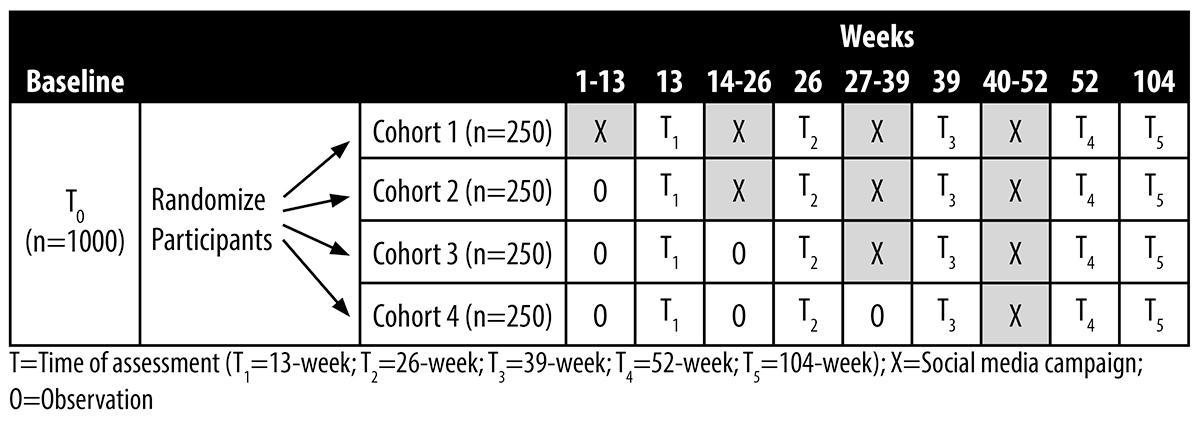
Randomized stepped-wedge trial design.

The cancer prevention social media campaign will be tested using a randomized stepped-wedge design (Figure 1).

Following baseline assessment (T_0_), emerging adults will be stratified by state, ethnicity (racial and ethnic minority individuals vs non-Hispanic White individuals), and biological sex (male vs female) and randomly assigned to 1 of 4 cohorts differing in intervention duration by the project biostatistician. All cohorts will complete postintervention assessments at weeks 13 (T_1_), 26 (T_2_), 39 (T_3_), 52 (T_4_), and 104 (T_5_). All data collection will be conducted using the secure web-based REDCap (Research Electronic Data Capture; Vanderbilt University) application. The primary outcomes measured at all assessment times will be MVPA, healthy eating patterns, nicotine product use, alcohol intake, sunburn, and HPV vaccination.

The social media campaign will be conducted over 12 months in 4 separate Facebook private groups. The intervention will start in each cohort at successive 13-week intervals, so cohorts will receive varying doses of campaign exposure (ie, 180 posts per 13-week interval) via stepped entry. Specifically, cohort 1 (250/1000, 25%) will start the social media campaign at week 1, receiving approximately 720 posts in 52 weeks. Cohort 2 (250/1000, 25%) will start the campaign at week 13 (approximately 540 posts), cohort 3 (250/1000, 25%) at week 26 (approximately 360 posts), and cohort 4 (250/1000, 25%) at week 39 (approximately 180 posts). Emerging adults will be told to read, react to, and comment on posts as often as they like. Exposure to cancer risk information and misinformation from other sources will be controlled by randomization.

The stepped-wedge quasi-experimental design was selected because it has several methodological advantages [91]. First, it allows for the random assignment of participants to 1 of the 4 cohorts, ensuring that potential confounding factors are evenly distributed across the groups. Each cohort is assigned to start the intervention at different times, creating a staggered, time-sequenced implementation schedule across the cohorts that creates the controlled nature of our trial. This design element allows for the within-study comparison of outcomes across different cohorts, with each cohort serving as a control for the others before they receive the intervention. Specifically, the control condition is represented by the periods when the cohorts have not yet started the intervention. For example, cohort 2 serves as a control group for cohort 1 before they commence their intervention in week 13. Second, the design also allows for comparisons between cohorts that have received the intervention for different durations. Third, by collecting data at multiple time points (T_1_-T_5_), the design facilitates the assessment of outcomes over an extended period that allows for the evaluation of trends and changes in outcomes over time within each cohort as well as comparisons between cohorts. The multiple baseline measures in cohorts 2, 3, and 4 will permit the examination of any trends or patterns before the intervention is introduced and will control threats to validity [92], as we can account for potential confounding factors, such as time-related effects or external factors, and compare cohorts at different stages of intervention exposure. This strengthens the internal validity of the study. Fourth, the design optimizes study resources by focusing on recruiting and retaining a longitudinal sample. This approach differs from securing a sample where half remain untreated, mitigating problems associated with control groups such as loss to follow-up, demoralization, disengagement, or noncomparability. It adheres to ethical standards by having all participants eventually receive the intervention but still preserves the controlled trial’s integrity.

### Cancer Prevention Social Media Campaign

The social media campaign will deliver 2 posts per day (n=approximately 720 posts) over 12 months, addressing cancer risk reduction, teaching digital and media literacy, encouraging family and friend communication, responding to misinformation, and highlighting community resources. The diverse content should be engaging and should avoid fatigue, but we will assess information overload and compare varying intervention durations (ie, 13, 26, 36, and 52 weeks).

#### Theoretical Approach to Social Media Intervention

We will adopt a multitheoretical approach to influencing rural emerging adults’ health behaviors. Social cognitive theory (SCT) [93] and self-determination theory (SDT) [94] will provide a framework and guide message development and measures of theoretical mediators, including cancer risk perceptions, self-efficacy and response efficacy, norms, social support, relatedness, autonomy, and motivation. SCT constructs address the environment (eg, environmental risks [UV exposure] and settings [health care access]), situation (eg, social norms of risk behaviors), behavior (eg, risk-reduction knowledge and skills), expectations (eg, good health outcomes), observational learning (eg, behavioral modeling), and self-efficacy (eg, confidence to perform prevention) [95]. Three needs must be satisfied when using SDT to foster well-being [94]: (1) competence—ability to control outcomes and feel self-efficacy, (2) relatedness—innate desire to interact with others, and (3) autonomy—need to be in charge of one’s life. Effective interventions implement techniques and strategies that support these 3 basic psychological needs. In need-supportive environments and contexts, extrinsic motivation can be transformed into higher-quality, intrinsic motivation, which is linked to positive health behavior change [96].

Posts will also include engagement strategies to encourage user-generated content and incorporate testimonials. The diffusion of innovations theory (DIT) and social network principles [97] explain that social media are uniquely influential because (1) user activity increases dissemination [98] and (2) information spread among social media communities, notably by knowledgeable peers, reduces uncertainty; makes sentiments dominant [65,99]; and motivates collective action [97,100] via social comparison [97,101] and collective identity that stabilizes actions [101]. Social media’s influence is further clarified by transportation theory [102]. According to this theory, personal stories can be more influential than didactic messages and expert advice [103] partly because of the audience’s identification with the characters [104]. Users often share stories on social media.

Digital and media literacy principles, including “active inquiry and critical thinking about mediated messages” [105], will be addressed in the campaign because many emerging adults are not critical media consumers [106]. Strategies to help emerging adults navigate the media landscape, combat echo chambers of misinformation, counterargue critical information, and develop trust in public health voices are imperative [107,108]. Fact-checking and corrections are important strategies for the campaign, but counternarratives, peer correction, factual elaboration, coherence and credibility appeals, media literacy [109-113], eHealth literacy [114], and advice on safe content sharing are also essential for the posts [69,115]. Media literacy interventions have helped health-related decisions and provided skills to combat unhealthy messages across health topics [116-120], suggesting they will make emerging adults “better prepared and willing to take preventive actions recommended by health professionals” [121]. We will convey how to identify media sources, their intentions, and misinformation; conduct web searches; overcome discomfort of new behaviors; converse with others about cancer risks (skills acquisition); highlight benefits to self, family, and friends (positive outcome expectations); and reinforce that they can reduce risks (self-efficacy). We will measure digital and media literacy skills and the level of misinformation (ie, holding inaccurate beliefs) about cancer prevention as mediators.

#### Social Media Posts

Social media posts will contain text with images, infographics, videos, and links to websites or other social media platforms (eg, Instagram and YouTube) from government, health care, news media, and trusted sources. Preferred health content for emerging adults of any gender and various social groups (eg, race, ethnicity, income, education, and living circumstances) will be identified through community engagement methods. Messages will address SCT [93], SDT [94], DIT [97], and media literacy principles [122] described earlier. The posts will be positive, at the seventh-grade reading level, and in English. The posts will cover 5 content areas:

- Cancer prevention: posts will address 6 cancer risk factors and various methods to improve them including behavioral skills for risk reduction, benefits of engaging in such behaviors, social support for these efforts, and strategies to minimize the social and financial costs associated with cancer risk reduction. In addition, advice from health care providers will be shared to help individuals overcome barriers to adopting healthier behaviors. Risk-reducing behaviors will be targeted by the posts include increasing MVPA and healthy eating behavior (more vegetables, fruits, and whole grains and less red and processed meats, sugar-sweetened beverages, highly processed foods, and refined grains); decreasing nicotine product use, alcohol intake, and UV exposure (sunburn); and increasing HPV vaccination. Posts will seek to improve several theoretical mediators, including self-efficacy and response efficacy and perceived risk, and to link cancer prevention to personal goals, including compatibility with values, observable benefits, and simplicity. Posts will also address cost of cancer prevention, present descriptive norms related to healthy and unhealthy behaviors, and highlight social support from family, friends, and partners. They will also promote emerging adults autonomy for their own health and decisions to adopt healthy behaviors. Posts will highlight the cancer prevention benefits of these behaviors and other benefits that may motivate emerging adults to adopt risk-reduction behaviors, including appearance (eg, avoiding skin aging), social (eg, reducing alcohol-induced partner violence), financial (eg, cost savings from quitting nicotine products), and disease prevention (eg, cardiovascular health) benefits. We will use a rotation pattern that covers the 6 cancer risk factors in at least 1 post per week and highlights 1 cancer risk factor in a series of posts each week to provide an in-depth intervention.
- Digital and media literacy skills: media literacy posts will focus on critical thinking, skill acquisition, and misinformation correction. Posts will aim to improve emerging adults’ digital and media literacy competencies related to (1) access, (2) analysis, (3) creation, (4) reflection, and (5) action [122]. Posts will focus on assessing message credibility and quality (eg, authorship [eg, bots], purpose [eg, marketing], construction, and algorithms) and validity (eg, original source identification, images, deep fake videos, and scientific evidence). Differences in storytelling and scientific evidence will be discussed. Marketing messages will be addressed because advertisers (eg, tobacco, alcohol, and tanning industries) reach emerging adults through social media promotions [41]. Even brief exposure to these marketing messages can instill positive attitudes toward the products [123].
- Responding to misinformation: we will use our best practices to respond to misinformation [124], that is, messages in conflict with scientific and medical information and advice. Misinformation will be identified by monitoring users’ reactions and comments, auditing the media landscape, and responding immediately [125] to forestall it from going viral. We will respond both proactively in the feed and reactively in replies to the comments. Responses will show empathic engagement and acknowledge users’ uncertainty, confusion, or motivations to prevent defensiveness and maintain trust [125]. Posts will then debunk misinformation by fact-checking, providing factual elaboration and coherence appeals with evidence-based sources [110,111], reframing information to fit existing beliefs, using resistance-to-persuasion tactics (2-sided appeals [126,127] and inoculation [128-130]), telling stories that offer personalized advice, presenting credible statistics and science [111,112,131,132], highlighting prevention actions by resistant groups [132], and depicting patient-provider interactions [132].
- Family and friend communication: emerging adults are expected to reside in several living arrangements, often with other adults, so posts will present prompts to talk with family (eg, parents, siblings, and partners) and friends about cancer prevention (and content from the campaign posts). Posts will focus on skills for active listening, self-disclosure, support, and conflict management.
- Referral to community resources: a website will be created that contains links to web-based tools and brief interventions to help emerging adults alter cancer risk behaviors, especially those that may be unknown to emerging adults. Examples include quit-smoking services, portals to state vaccination records, tools for managing the multidose HPV vaccination schedule, and fact sheets and guidelines from health authorities. Resources will be identified by the community advisers and maintained by the investigators. A link to this website and its relevant resources will be included regularly in posts.

#### Development of Cancer Prevention Posts

An agile, just-in-time process will be used to create and adapt posts to be responsive to emerging adults and to reflect current events [133]. Investigators will prepare a campaign framework for developing posts, identifying target behaviors for each cancer risk factor and key theoretical principles. Project staff will continuously audit cancer prevention information and misinformation in (1) published literature, government reports, and national surveys; (2) participant comments on posts; (3) quarterly web-based emerging adult focus groups; and (4) advisory board input. The investigators and media developers will revise posts, add emerging themes, and tailor posts to key subgroups. Initially, 3 months of campaign posts will be prepared, with additional posts developed during the campaign, creating a planned adaptive campaign. Social media posts will be written in English because rural emerging adults are the primary target group, with Hispanic and American Indian emerging adults comprising a minority of them. English continues to be the most common language on the internet, especially in the United States [134,135]. Nearly all young adults in the United States are proficient in English, even most foreign-born young adults [136].

To ensure campaign exposure, posts must regularly engage users because the Facebook algorithm presents posts from the private groups more frequently and prominently in participants’ newsfeeds when they engage more with the group’s posts. To achieve high visibility and engagement, we will post twice per day and (1) provide novel, high-interest, useful, and current-event content; (2) adjust for season; (3) link to content from other emerging adults to create descriptive norms; (4) address age differences and cultural barriers and facilitators; (5) use ethnically diverse emerging adult images that can improve health communication [137]; and (6) use formats such as stories, polls, questions and answers, videos, and visuals, and invite comments [58,98,138-141].

The campaign will be pilot-tested with rural emerging adults (N=25) meeting the inclusion and exclusion criteria displayed in Textbox 1 (13/25, 52% female individuals and ethnically diverse). It will contain a feed of 56 campaign posts (2 per day) over 4 weeks, and participants will provide feedback in focus group discussions.

#### Implementation of Social Media Campaign

The social media campaign will be implemented through Facebook private groups. A staff person will serve as the community manager and schedule posts twice a day on all 7 days of the week (1 message in the morning and 1 in the afternoon) to achieve reach [142,143]. In each cohort, emerging adults will receive welcome posts on purpose and ground rules (eg, respect for others) and then receive the ongoing feed delivered in their separate private group so that posts are identical across cohorts, timely, linked to current events and news stories, relevant for seasons, and engaging. We will not start each cohort at the beginning of the feed because it would require adjusting earlier posts to be current, making them dissimilar across cohorts. The community manager will monitor reactions and comments from emerging adults, answer questions, address uncertainty, and correct misinformation [58,124,133]. The community manager will promote peer influence by, for example, (1) recruiting high-frequency emerging adult users to be guest moderators for up to 3 days to schedule posts and reply to comments and (2) hosting Facebook Live events with emerging adult experts (eg, an emerging adult dietitian). If any bullying arises, the community manager will de-escalate it by (1) highlighting empathy and (2) sending direct messages to stop it. If participants leave a group, they will be contacted to see why. Project staff, except the community manager and the project coordinator, will be blinded to the cohort membership.

Facebook’s private group function possesses unique features not found in other social media platforms. These distinctive qualities offer practical methodological advantages and enhance experimental rigor. Facebook remains one of the most popular social media platforms for emerging adults, used by 70% of the population nationally (among which 70% use it daily), with the majority reporting use in nearly all demographic groups, including by age, rurality, race, ethnicity, and income [43]. Thus, nearly all emerging adults will have existing accounts and be familiar with its interface. Other social media place limits on content delivery that would restrict cancer prevention messaging, including by restricting format (eg, YouTube and Instagram mainly use video and images), length (Snapchat and TikTok deliver short videos), and permanence (Snapchat posts last for 24 hours after posting), and they all restrict the ability to link to other web content more than Facebook. Facebook’s private group function will also help ensure that posts appear in users’ feed and will limit access to posts to group members (posts cannot be shared on other social media) to avoid contamination. Finally, Facebook has superior data analytics for tracking exposure to content compared with other platforms.

### Community Engagement Methods

Community-based participatory research methods will inform the study and cancer prevention campaign. Partnership processes aligned with the model by Sandoval et al [144] will provide a framework for the study: (1) knowledge of *contexts* that inform catchment area needs; (2) culturally informed *partnership processes* guiding engagement; (3) *intervention and research* protocols responsive to rural, low-income, and underserved conditions; and (4) participatory *outcomes* disseminated to partners. The research team will draw on the community networks of the Four Corners Cancer Centers Collaborative through each cancer center’s Community Outreach and Engagement program [145] to convene 2 community advisory boards for the project—an Emerging Adult Advisory Board (EAAB) and a Stakeholder Organization Advisory Board (SOAB). Up to 16 emerging adults from rural counties in the Four Corners states, diverse in gender and ethnicity, will be recruited to serve on the EAAB. The EAAB will meet quarterly with investigators in years 2 and 3 and biannually in years 1, 4, and 5. Members provide input on the social media campaign and implementation protocols (year 1) and review proposed posts (years 2 to 3), with attention to the relevance of posts for the circumstances experienced by rural emerging adults, especially the challenges experienced by low-income and marginalized emerging adults. Up to 4 stakeholder organizations that provide health promotion and cancer prevention services to rural emerging adults in each of the 4 states will be recruited for the SOAB. The SOAB will meet twice annually to review messaging, identify local resources, and plan dissemination efforts. Both advisory boards will advise on trial findings and dissemination efforts in years 4 to 5.

A series of focus groups with up to 8 rural emerging adults per group, meeting the inclusion and exclusion criteria (Textbox 1), will be conducted during the project to help develop the social media campaign. Discussions will cover cancer risk behaviors, social media use, health information seeking, misinformation, the context of health behaviors, and current issues and trends. Emerging adults will review social media posts and suggest how to engage rural emerging adults and reflect local contexts and issues. The results will be summarized and used to adapt posts to be responsive, timely, and engaging for emerging adults and targeted to key subgroups.

### Measures

#### Primary Outcomes: Cancer Risk Behavior Outcome Measures

We will use validated self-report measures of each cancer risk behavior in the T_0_-T_5_ surveys presented in Table 1.

**Table 1:** Primary and secondary cancer risk behavior outcome measures.

Cancer risk behavior	Measure	Metric
Physical activity	Primary and secondary: Global Physical Activity Questionnaire [146]	Minutes per week of MVPA^a^ (primary) Meet 150 minutes per week goal (secondary)
Diet	Primary: Dietary Screener Questionnaire [147,148] Secondary: other meal behaviors [149-152]	Intake per day of fruits, vegetables, whole grains or fiber, added sugars (from sugar-sweetened beverages), and red or processed meats Frequency of eating meals and snacks, fast food, and skipping meals
Nicotine product use	Primary: 30-day prolonged tobacco or nicotine product abstinence [153] Secondary: 7-day point prevalence of smoking [153] Secondary: readiness to quit [154]	Use in the past 30 days (every day, some days, and not at all) Use in the past 7 days (every day, some days, and not at all) 10-point rating (1=no thought of quitting and 10=taking action to quit)
Alcohol intake	Primary: consumption of alcoholic drinks [155] Secondary: binge drinking (male individuals: 5 drinks per sitting and female individuals: 3 drinks per sitting)	Number of days in the past 30 days; number of drinks per occasion Number of times in the past 30 days
UV exposure	Primary: sunburn prevalence [156] Secondary: sun protection behavior [157]	Number of sunburns Percentage of sun exposure days using sun protection
HPV^b^ vaccination	Primary: any dose of HPV vaccine [158] Secondary: completion of vaccine series	Received 1 or more doses Received 2 or 3 doses as recommended for age

^a^MVPA: moderate to vigorous physical activity.

^b^HPV: human papillomavirus.

Self-report measures are most practical for this pragmatic trial, with the large sample of emerging adults in 4 geographically large states (approximately 1,100,700 sq km), web-based recruitment, and a multirisk factor approach that makes clinical and observational measures infeasible. The recall period for nicotine abstinence, alcohol use, and sunburn prevalence measures will be 30 days, which improves reliability [156]. Physical activity and other meal behaviors will be measured for the past 7 days, and HPV vaccination will be measured from baseline.

The primary outcome measures of physical activity, diet, and nicotine abstinence will be validated in subsamples of emerging adult participants at baseline and at the 52-week posttest stage. Physical activity and diet self-report measures will be verified with (1) accelerometry (ActiGraph GT9X) [159,160] and (2) 24-hour recalls (for 3 random days; 2 weekdays and 1 weekend day) [161] in a subsample of emerging adults (139/1000, 13.9%) at baseline and repeated at 52 weeks. Nicotine abstinence self-report measures will be verified via saliva cotinine assays (Salimetrics assay) on a subsample of 15% emerging adults reporting abstinence at 52 weeks [153]. Objective measurement of alcohol use, sunburn, and HPV vaccination is infeasible within the scope of the trial.

#### Mediators

Mediators will be assessed in all surveys. These include theoretical antecedents—cancer risk (SCT; severity and susceptibility: 6 items); self-efficacy and response efficacy (SCT) [162]; cost of cancer prevention [162]; descriptive norms (SCT; 2 items; prevalence among people you know and 5 people you know best) [163]; social support from family, friends, and partners (SCT); relatedness (SDT; 4 items) [164]; autonomy and motivation (SDT; 10-point contemplation ladder) [154,165]—family and friend communication about cancer prevention (SDT and DIT; if emerging adults shared information from feed with family and friends) [166]; and vaccine antecedents (ie, confidence, constraints, complacency, calculation, and collective responsibility [167]). Digital and media literacy will be assessed with 3 competency measures—self-perceived media literacy (4 items) [168], perceived social media literacy (6 items) [169], and eHealth literacy (8 items) [170]—along with cancer prevention misinformation (8 accurate and 8 inaccurate Likert statements recoded for belief in misinformation).

#### Potential Covariates

The following variables, measured at baseline, will be assessed as covariates:

- Participant characteristics: race, Hispanic ethnicity, gender identity, biological sex, RUCC codes (4 to 9), age, education, employment, and emerging adults’ height and weight for BMI (treated as a covariate because of stability at this age) [171]
- Household features: marital status, parenting status (children at home), household composition, food insufficiency [149], and use of government nutrition assistance programs [155]
- Health care use: insurance status [172] and prior visit to a physician for routine preventive care
- Cancer history: personal and family history of cancer
- Cancer messaging: exposure to cancer information (ie, topics and sources [health care provider, social media, website, news media, and conversations]) [64] and perceived credibility of various media [133]
- Social desirability: a socially desirable response set 5-item measure [173] to account for socially favorable response bias.

#### Social Media Campaign Engagement Measures

Behavioral and experiential measures of campaign engagement [174,175] will be collected, guided by the model of engagement by Perski et al [176]. Behavioral measures will be (1) staff records of posts; (2) counts of emerging adults’ views, reactions (eg, like or sad), and comments extracted in identified format using our custom-written app and coded for content and pro, anti, or neutral sentiment by 2 trained research assistants [113]; and (3) use of resources on the project website recorded by the web server. Experiential measures will be collected in each postintervention test, including time spent and frequency of reading posts, flow experience (ie, social interaction, enjoyment, and concentration) [177], cancer information overload [178], and sharing post content with others.

### Statistical Analysis Plan

#### Hypothesis Testing

We plan to use advanced statistical methods suitable for stepped-wedge designs to compare outcomes between different cohorts and time points, which will account for both time effects and intervention effects. This includes using mixed-effects models or generalized estimating equations that can adjust for time-related trends and cohort effects. In addition, by comparing cohorts that start the intervention at different times, we will isolate the effect of our campaign from other external factors and assess whether changes in the cancer risk behaviors are more pronounced or accelerated following the introduction of our intervention compared with the periods before the intervention. In addition, our analysis will adjust for potential confounding factors that might influence cancer risk behaviors, such as age, sex, ethnicity, and other relevant sociodemographic factors. This approach ensures that the control aspects of the design are rigorously analyzed.

Specifically, the 2 hypotheses and exploratory research questions will be tested using R (R Foundation for Statistical Computing) [179] and Mplus, V8.2 (Munthén & Munthén) [180], a structural equation modeling program that allows for growth models and latent constructs (to model measurement error appropriately), repeated measures, direct and indirect effects, and moderators using interaction terms and multiple group analysis [181]. Mplus will handle missing data via full information maximum likelihood. All tests will be intent to treat. To mitigate false discovery (type I error), an α of .008 will be used (ie, the traditional α=.05÷6 [number of cancer risk factors considered]) [182]. Once the full sample is recruited, baseline data will be described and plotted, measurement models will be assessed, and transformations for normality will be examined and applied.

The effect of the treatment on each of the primary outcomes will be examined using a linear mixed-effects model for a stepped-wedge design, as outlined by Hussey and Hughes [183] and Li et al [184]. Repeated measures (6 per individual—T_0_, T_1_, T_2_, T_3_, T_4_, and T_5_ in Figure 1) will be regressed on time since the start of the study (“calendar time”) and time since the start of message exposure (“exposure time”), both of which are expressed as categorical variables via dummy codes. Any mediators found to be significantly impacted by the treatment will be subsequently examined as mediators of the treatment effect on the outcomes via formal mediation models. Multilevel mediation models will be fit as described by Preacher [185] using the structural equation modeling program. As moderation tests require a vastly larger sample than tests of main effects [186], we will examine them without conducting null hypothesis significance tests [187]. We will estimate each model, evaluate the effect magnitude as a function of the moderator, construct bootstrap CIs to illustrate uncertainty, and use false discovery rate controls [182,187]. Linear mixed-effects models for hypothesis 1 will be extended to consider effect modification because of cancer risk behaviors, campaign exposure variables, biological sex, and the time of year of data collection (to control for seasonal fluctuations in UV levels, food availability, and alcohol intake).

Finally, behavioral and experiential engagement will be tested for campaign dose response (ie, duration of the campaign) related to cancer risk behaviors. In addition, behavioral engagement with posts (ie, views, reactions, and comments) on certain topics (eg, risk behaviors, media literacy, or family communication) and formats (eg, text, video, or interactive features such as polls) will be examined to determine if they have an impact on campaign effectiveness.

#### Power Analysis

To determine the appropriate sample size for the stepped-wedge design, we used the Shiny Cluster Randomized Trial calculator [188], setting α at .008 and assuming, conservatively, that the within-person correlation of the repeated measures is 0.5. Power analysis focused on the main effect on outcomes at the 104-week posttest score being significantly better than preintervention scores (ie, the time-averaged intervention effect). We considered a mean standardized difference between pre- and postexposure measure of 0.2 for a continuous outcome and a difference in pre- versus postexposure prevalence of 0.40 versus 0.48 for a binary outcome. These are conservative and relatively small effects based on our past assessment of a social media campaign [166,189] and the expectation that emerging adults are unlikely to see all posts. We will achieve a power of 0.80 with 115 people per cohort for a continuous outcome and 175 per cohort for a binary outcome. We have planned for a 30% (300/1000) dropout rate, so we will enroll 1000 emerging adult participants and expect to finish the trial with approximately 175 individuals in each cohort. We will use full information maximum likelihood methods for estimation; thus, even incomplete cases will be retained.

### Ethical Considerations

The WCG Institutional Review Board reviewed and approved the protocols for the research (study #20223673). Participation will be voluntary, and participants will read and sign an informed consent form approved by the institutional review board. The consent form will present the purpose of the research, the procedures, known risks and benefits, and the use and security of the data. All data collected in the study will be confidential, and participant identity will not be disclosed publicly. Emerging adults that participate in a focus group discussion will be compensated US $40. In the stepped-wedge design, participants will be paid US $30 for baseline, US $15 for 13-week, US $15 for 26-week, US $15 for 39-week, US $30 for 52-week, and US $30 for 104-week postintervention tests (US $135 in total). Those who are selected for the verification of outcome measures via accelerometry, 24-hour dietary recalls, and saliva cotinine assays will receive US $25 compensation for each measure.

## Results

### Project Initiation and Administration

The study commenced in September 2022. It is supervised by a steering committee comprising the project principal investigators (D Buller and A Sussman) and coinvestigators from each site. The steering committee is meeting once a month to make design and administration decisions, plan activities, track progress, troubleshoot problems, maintain consistency in actions, implement quality controls, and communicate with the funding agency and institutional review board. In addition, the project staff at each site meet monthly with the coordinating center lead staff to ensure timely and regular communication and timeline adherence.

**Figure 2:**
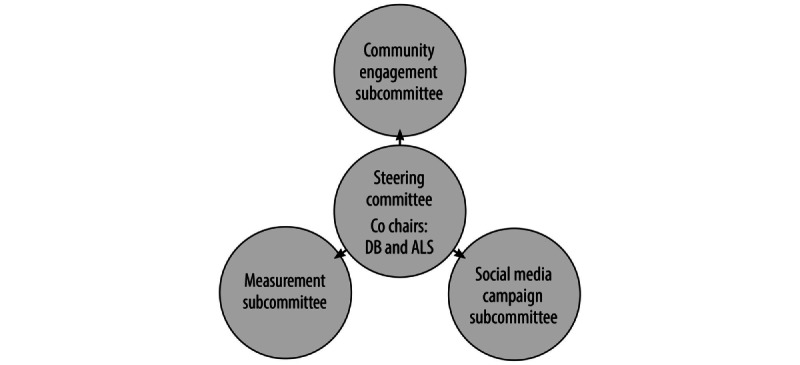
Steering committee and subcommittees for project management.

Project activities are being conducted by 3 subcommittees of investigators and project staff, meeting bimonthly (Figure 2). The Community Engagement Subcommittee is managing the partnerships with emerging adults and key stakeholder organizations in the rural counties of the Four Corners states. Advisory boards with members of these 2 groups have been convened and meet regularly with the research team. The Social Media Campaign Subcommittee is developing campaign content and implementation procedures. It has created a framework and procedures for creating social media posts and protocols for the community manager and responding to misinformation. The Measurement Subcommittee has identified assessment instruments, is piloting the measures with samples of emerging adults, and is developing data collection and retention procedures. A subcommittee guiding statistical analysis will be created in the future once the trial commences. The steering committee is functioning as a working group on trial recruitment issues and procedures.

### Project Advisory Boards Meetings

The EAAB and SOAB have been formed with members recruited from each of the 4 states and met with the project investigators and staff in 2023. The EAAB members (3/8, 38% from Arizona, 2/8, 25% from Colorado, 1/8, 12% from New Mexico, and 2/8, 25% from Utah) are diverse in gender (6/8, 75% women and 2/8, 25% men), race and ethnicity (4/8, 50% White, 3/8, 38% American Indian, and 3/8, 38% Hispanic), and age (mean 22.4 years, SD 2.0; range 19-25 years). Nearly all (7/8, 88%) are students (only 1/8, 12% was employed for wages), but 62% (5/8) of the EAAB members have ≥4 years of college education. In addition, 12% (1/8) of the members live alone, 25% (2/8) of the members live with parents, 38% (3/8) of the members live with other people (not with family), and 25% (2/8) of the members live in a college dormitory. The SOAB members represent a diversity of organizations serving the health and education needs of rural counties, specifically emerging adults. Advisers self-identify as female and White (2/14, 20% were Hispanic) and have a mean age of 49.9, SD 9.1 (range 41-63) years, and all completed some college education, with most having a ≥4-year degree education. Advisory boards will meet twice a year (with the EAAB meeting quarterly during project years 2 and 3) and also communicate through Facebook groups to provide ongoing input.

The initial advisory board meeting agendas focused on introducing the project and discussing important health issues for emerging adults, social media use, misinformation on social media, and community resources for emerging adults (SOAB only). In the EAAB, participants identified nicotine product use, healthy eating, and alcohol intake as the most important health priority areas for emerging adults. The emerging adults described a passive approach to reviewing health information on social media, noting that they do not routinely initiate a search but instead encounter a high volume of posts. Furthermore, members of the EAAB were generally confident in their ability to discern the trustworthiness of the posted health information. The EAAB reviewed initially created posts on cancer risk factor reduction, providing input on text, visuals, and links to outside sources. The SOAB cited the importance of HPV vaccination, alcohol intake, and healthy eating as major health priorities for emerging adults among the 6 cancer risk factors. Most organizations represented on the SOAB use social media to reach emerging adults. The SOAB members expressed a higher degree of concern compared with emerging adults in determining the trustworthiness of posted health information. Finally, most SOAB members indicated that there are not enough community resources to address the complete list of risk factors included in our study. Subsequent EAAB and SOAB meetings will deepen the exploration of these issues.

### Social Media Campaign Framework Development

A framework has been created to guide the development of social media posts. It contains information that will be used to track messages by topic, primary and secondary outcomes, theoretical mediators, communication mediators, media literacy mediators, message design features, and engagement techniques. The message text, corresponding link, and image or video used in the post will also be tracked. In addition, the date and time the post is published to the Facebook feed and the Facebook link for the message will be recorded. Investigators with content expertise in each of the 6 cancer risk behaviors have identified key precursor behaviors and effective past interventions, especially with young adults, to incorporate into the framework. In addition, specific protocols were developed for the community manager, who will administer the social media campaign, the process of responding to misinformation during the campaign, and the process for the research team to review messages before posting to the social media feed. A style guide for the post content is also in development.

## Discussion

### Principal Findings

Emerging adulthood is an important period for the promotion and sustainment of guideline-recommended cancer prevention behaviors. Many rural emerging adults experience several cancer risk factors, but successful interventions for them are lacking. Emerging adults in the rural areas of the Four Corners states may be at a particularly high risk of adopting unhealthy cancer-preventive behaviors. With the popularity of web-based resources for emerging adults, an innovative multirisk factor intervention over social media should be able to reach this underserved population and reduce emerging adults’ cancer risk behaviors. Web-based health programs have been effective in past research [46,64,190-197]. In particular, social media has influenced cancer risk behaviors in some past studies [47-57], although studies evaluating social media with only emerging adults are uncommon, as are prospective randomized study designs [83,198-203].

### Strengths and Limitations

The planned trial has several methodological strengths, including the unique multi-institutional team of investigators with expertise in each of the health behaviors of interest as well as cancer prevention and control; a diverse, rural population; an understudied age group; extensive community engagement; a multirisk factor approach; the use of social media; a rigorous study design; and high dissemination potential. The stepped-wedge design will provide experimental control, reduce error variance, avoid problems with control groups, model campaign dose, and efficiently use resources to recruit and retain the sample. We will recruit from an internet panel rather than from the community to obtain a large sample from the sparsely populated Mountain West. The social media campaign will be based on theories of health behavior change and social media influence. The stepped-wedge design and a multifaceted analysis of campaign engagement will be used to assess campaign dose effects.

Several design decisions were made to avoid or reduce potential weaknesses. Using a research panel to improve sample diversity is appropriate for a field experiment that does not aim to estimate population prevalence [204]. Research panel members may participate in multiple studies, but this does not appear to cause low-quality results [205]. Social media advertising was considered for delivering the intervention, but it is difficult to achieve advertising exposure [206] and control contamination, and any test would require a very large sample. Emerging adults with a history of cancer will be included because (1) their numbers are small [207], (2) many engage in cancer risk behaviors [208] and can benefit, and (3) excluding them would reduce generalizability. Self-report measures can contain errors; however, given the large geography and virtual environment, they are practical. We have selected validated self-report measures and will verify physical activity, diet, and nicotine abstinence measures using a subgroup validation cohort that provides more rigorously measured health behavior data. The primary evaluation will test the impact of the overall social media campaign, not the individual message strategies. To obtain insights on which strategies impact outcomes, we will examine campaign engagement measures, theoretical mediators, and the association of message topic and format with changes in cancer risk behaviors and theoretical mediators. Although most emerging adults engage with a variety of social media platforms, we have chosen to use the Facebook platform for delivering our social media campaign. This decision is based on its practicality and widespread use among emerging adults as well as its features that enhance experimental rigor.

### Conclusions

New strategies are needed to improve public health information dissemination, correct misinformation [66-68], and promote skills to help emerging adults judge the veracity of web-based content to promote cancer risk reduction. The study will provide this innovation in several ways. A unique, theory-based social media campaign will be created that delivers relevant, credible, and timely content on reducing multiple cancer risks among emerging adults and can be translated to emerging adults in other rural regions. It promotes the reduction of cancer risk behaviors in the diverse (based on ethnicity and education) emerging adult population in the Four Corners area, which has been largely overlooked in past research. This will be one of the first studies using participatory strategies to focus an intervention on behavioral and environmental cancer risk factors and health disparities in an emerging adult population. Emerging strategies will be used to correct misinformation about cancer risk behaviors on social media, along with promoting digital and media literacy skills to emerging adults. The study should have a major impact on emerging adults’ cancer risk behavior decisions and the consumption of accurate cancer information. Finally, the findings should be applicable to other cancer communications and disease prevention efforts for rural emerging adults.

## Abbreviations

DIT: diffusion of innovations theory
EAAB: Emerging Adult Advisory Board
HPV: human papillomavirus
MVPA: moderate to vigorous physical activity
REDCap: Research Electronic Data Capture
RUCC: Rural-Urban Continuum Codes
SCT: social cognitive theory
SDT: self-determination theory
SOAB: Stakeholder Organization Advisory Board

## Data Availability

The data collected in this study will be made available in a publicly accessible data repository at the conclusion of the research.
